# Advancing sustainable healthcare through multidisciplinary stroke team rehabilitation

**DOI:** 10.3389/fstro.2024.1509831

**Published:** 2025-01-09

**Authors:** Hanna C. Persson, Malin Reinholdsson, Elvira Lange, Stefi Barna, Annie Palstam

**Affiliations:** ^1^Rehabilitation Medicine, Institute of Neuroscience and Physiology, Department of Clinical Neuroscience Sahlgrenska Academy, University of Gothenburg, Gothenburg, Sweden; ^2^Department of Occupational Therapy and Physiotherapy, Sahlgrenska University Hospital, Gothenburg, Sweden; ^3^Research, Education, Development and Innovation, Primary Care, Region Västra Götaland, Vänersborg, Sweden; ^4^Department of Health and Rehabilitation, Institute of Neuroscience and Physiology, Sahlgrenska Academy, University of Gothenburg, Gothenburg, Sweden; ^5^General Practice/Family Medicine, Department of Public Health and Community Medicine, Institute of Medicine, The Sahlgrenska Academy, University of Gothenburg, Gothenburg, Sweden; ^6^The Centre for Sustainable Healthcare, Oxford, United Kingdom; ^7^Department of Rehabilitation Medicine, Sahlgrenska University Hospital, Gothenburg, Sweden; ^8^School of Health and Welfare, Dalarna University, Falun, Sweden

**Keywords:** planetary health, carbon footprint, rehabilitation, sustainable healthcare, sustainable development, environmental sustainability

## Abstract

Empirical studies evaluating stroke team rehabilitation interventions from a sustainability perspective are scarce. This paper highlights the significant role of multidisciplinary stroke team rehabilitation in promoting sustainable healthcare by applying principles of sustainable healthcare. Climate change and air pollution are significant risk factors for stroke and other cardiovascular diseases. Healthcare contributes to 5% of global CO_2_ emissions, exacerbating the disease burden associated with climate change. The vulnerability of individuals with disabilities to climate change has been highlighted, calling for global collaboration to address climate justice and health equity. This paper argues that multidisciplinary stroke team rehabilitation is essential for achieving sustainable stroke care, optimizing patient functioning, and contributing to all principles of sustainable healthcare: prevention, patient empowerment, lean pathways, low carbon alternatives, and efficient resource use. Timely assessments and dose-specific interventions are crucial for successful outcomes, providing significant co-benefits for healthcare resource use. Enhancing self-management and patient empowerment reduces healthcare utilization without compromising health outcomes. Telerehabilitation increases accessibility to healthcare services, particularly where transportation is challenging, and complements hospital-based procedures. Preventive healthcare activities, with their low carbon footprint, offer strong incentives for optimizing secondary prevention in stroke. Overall, multidisciplinary stroke team rehabilitation aligns with all sustainable healthcare principles, reducing overall healthcare consumption through optimized functioning and health. Increased investment in rehabilitation resources leads to better quality of care and reduced long-term resource use. By integrating sustainable practices, stroke team rehabilitation can significantly contribute to sustainable healthcare, addressing both human and planetary health.

## Introduction

Empirical studies evaluating stroke team rehabilitation interventions from a sustainable healthcare perspective are lacking. To our knowledge, only one study in stroke rehabilitation has included evaluation of environmental, economic and social aspects of sustainability (Mortimer et al., 2018). This paper argues that multidisciplinary stroke team rehabilitation is a crucial component of sustainable healthcare, aligning with key principles of prevention, patient empowerment, lean pathways, low-carbon alternatives, and efficient resource use.

### Climate change and health in stroke—Call for action

Interventions to mitigate climate change and reduce environmental pollution are a high priority in stroke prevention and care, as stated in the European Stroke Organization (ESO) Action Plan (Norrving et al., 2018), due to the increasing evidence of climate change and air pollution as significant risk factors for stroke and other cardiovascular diseases (Bejot et al., 2018; Cohen et al., 2017; Ljungman et al., 2019; Verhoeven et al., 2021; Yuan et al., 2019). Climate change, biodiversity loss, and pollution of air, land and water are consequences of human activities that fundamentally undermine the environmental conditions that support human life on earth (Watts et al., 2015). Further, International Society of Physical and Rehabilitation Medicine (ISPRM) has emphasized the explicit impacts of climate change in persons living with disabilities, acknowledging their specific vulnerability to the consequences from climate change, both concerning the ability to manage acute climate related catastrophes as well as to manage rehabilitation and health in a changed climate. The ISPRM call for rehabilitation providers to collaborate globally to address climate justice and health equity [(The International Society of Physical and Rehabilitation Medicine (ISPRM), 2023)]. At the same time, healthcare is a major contributor of air- and water pollution as well as greenhouse gas emissions, producing up to 5% of the total CO_2_ emissions globally, in parity with the emissions produced by the worlds' largest countries (Karliner et al., 2019), thereby exacerbating the disease burden associated with climate change (Chen-Xu et al., 2024).

The 2030 Agenda for Sustainable Development, which includes the Sustainable Development Goals, was adopted by all UN member states in 2015, calling for the mobilization of all countries, all stakeholders, and all people to work toward a more sustainable future (Jha et al., 2016). In this mobilization, healthcare has an important role to play, and 50 countries so far have committed to creating climate-resilient, low carbon, sustainable health systems, including 14 countries that have set a target date of reaching net zero emissions from healthcare by not later than 2050 (NHS England, 2020; Wise, 2021). In response to the 2030 Agenda, the World Health Organization (WHO) acknowledges the increasing demands for rehabilitation services to meet the current trends of aging populations and increasing number of people living with disability (World Health Organization, 2019).

While climate change is the largest health threat of this century, it also presents the greatest opportunity for healthcare professionals and departments to drastically reduce carbon use, whilst meeting equity targets and improving health outcomes, for the sake of present and future health of patients and populations (Hamilton et al., 2021).

Stroke team rehabilitation starts in the stroke unit. Stroke unit treatment increases survival and optimizes level of functioning after acute stroke (Stroke Unit Trialists, 2013). At a stroke unit, the multidisciplinary stroke team provides coordinated treatment, care and rehabilitation based on current evidence and best practice, corresponding to the needs of the patient (Langhorne et al., 2020). The multidisciplinary stroke team includes all disciplines required for acute stroke management, including radiology, and has been found to lead to a more efficient and rapid management (Norrving et al., 2018). Functioning is a key indicator for rehabilitation and has been suggested as a third health indicator, complementing the established indicators of mortality and morbidity (Stucki and Bickenbach, 2017). Further, it is argued that a focus shift toward functioning in health evaluations in the global health community could enable the attainment of sustainable development goals for health and wellbeing (Boggs et al., 2021).

**Figure 1:**
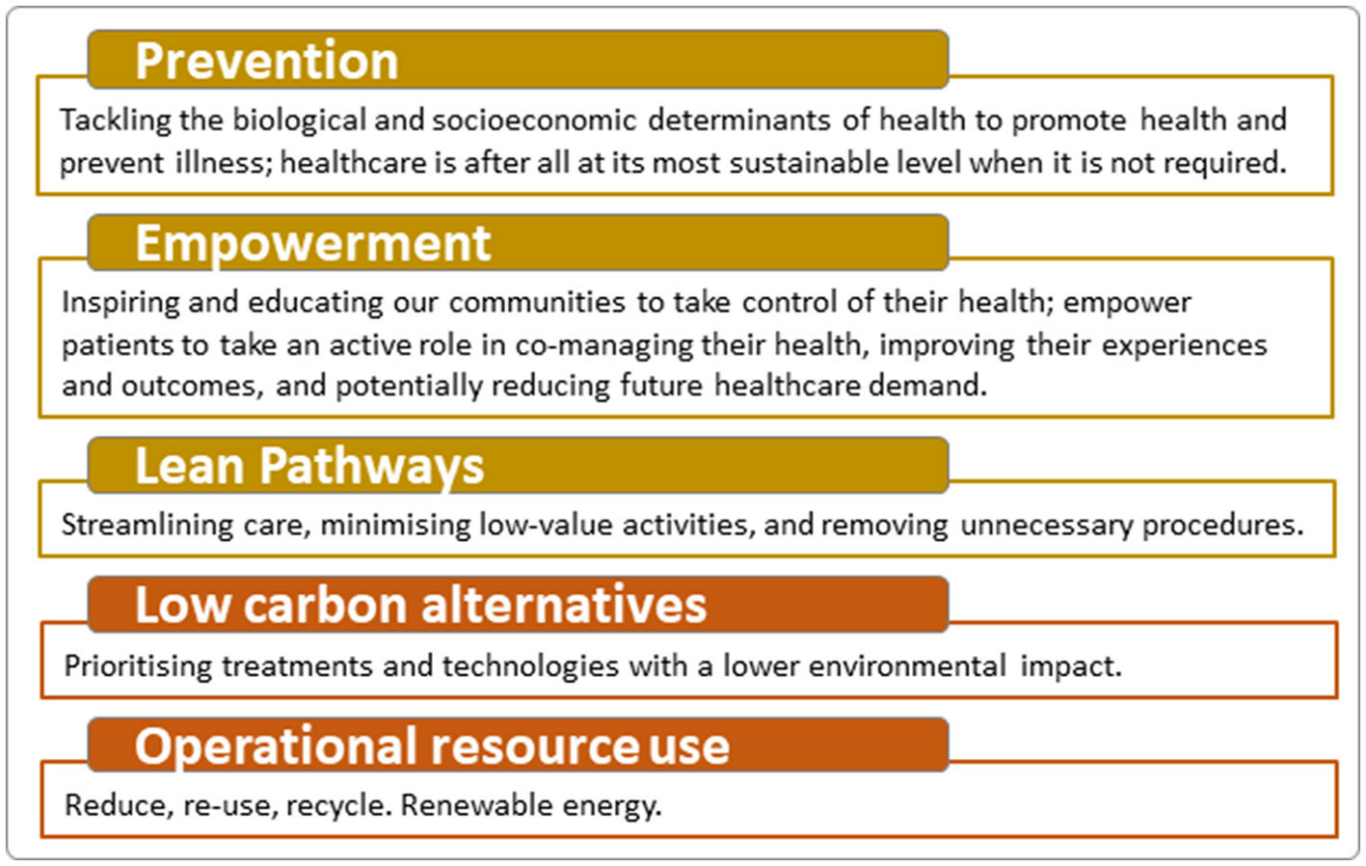
Principles for sustainable healthcare. Modified with permission from Mortimer et al. (2018).

Sustainable healthcare provides high-quality healthcare to meet the present needs of patients and populations without compromising the ability to meet future needs (Mortimer et al., 2018). Sustainable healthcare involves a holistic strategy for health services that effectively balances high-quality care, efficient use of resources, and minimal environmental impact, while ensuring long-term economic and social viability (Mortimer, 2010). Efforts to reduce the negative contribution of healthcare activities to climate change, and to create resilience to respond to the worst impacts of a warming climate, also offers an unprecedented opportunity to protect the health of patients, populations, and the planet (World Health Organization, 2015). A useful tool for clinicians is the sustainable value equation, that weighs patient outcomes against a “triple bottom line” of financial, as well as environmental and social impacts in their clinical decisions (Mortimer et al., 2018). Financial impact can be calculated by asking whether the desired outcome is affordable in the present as well as in future (Cadilhac et al., 2020). Environmental costs attempt to measure and internalize the impact on the natural environment from healthcare activities (Taylor and Mackie, 2017). Social impacts consider whether the intervention is accessible to all and consider whether hospital activities that help individual patients also facilitate or undermine the health of families, carers, staff, the local community, and vulnerable groups (Mortimer et al., 2018). Mortimer et al.'s (2018) model (Figure 1) for a transformation of clinical practice offers five principles for planning interventions in sustainable healthcare practice. This model emphasizes the importance of integrating environmental sustainability into clinical decision-making and highlights the role of healthcare professionals in promoting sustainable practices. By adopting these principles, multidisciplinary teams can enhance the overall sustainability of healthcare systems, ensuring that interventions are both effective and environmentally responsible. In this perspective paper, these principles are used as a framework to identify contributions of multidisciplinary stroke team rehabilitation to sustainable healthcare.

## The role of multidisciplinary stroke team rehabilitation for sustainable healthcare

**Figure 2:**
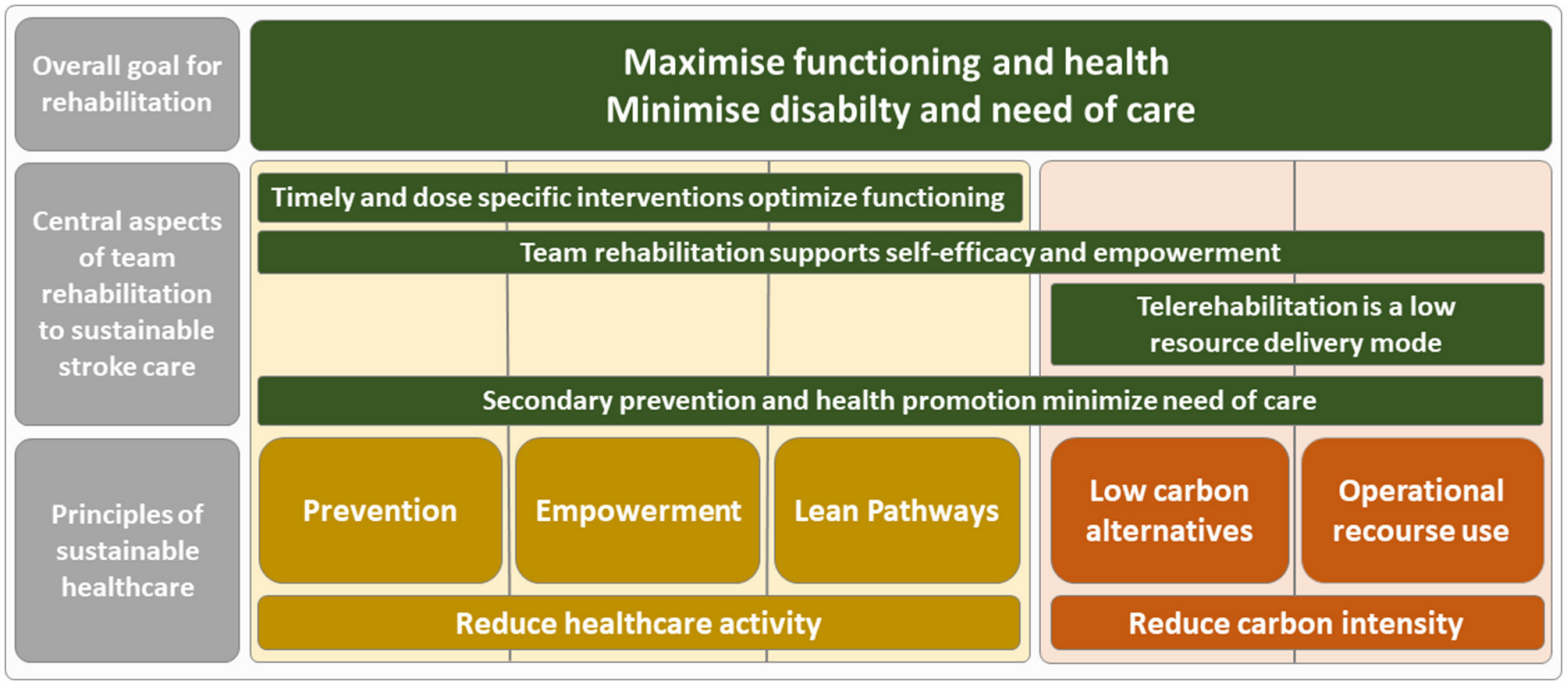
Aspects where multidisciplinary stroke team rehabilitation contributes to principles of sustainable healthcare.

We identified four central aspects in which stroke team rehabilitation contributes to principles of sustainable healthcare; *the provision of timely and dose specific interventions optimize functioning, team rehabilitation supports self-efficacy and empowerment, telerehabilitation as a low resource delivery mode*, and *secondary prevention and health promotion minimize need of care* (Figure 2).

The first central aspect of how multidisciplinary stroke team rehabilitation contributes to sustainable healthcare is through timely and dose specific interventions that optimize functioning. One example is early supported discharge (ESD) from hospital (Langhorne et al., 2017), where the stroke team provides rehabilitation at the patient's home. The ESD can decrease the length of hospital stay (Jee et al., 2022) and be a cost-effective alternative to in-hospital stroke rehabilitation (Anderson et al., 2002; Candio et al., 2022). Although not evaluated in research on stroke rehabilitation, the reduced need for in-hospital care means a decrease in environmental impact from healthcare since in-hospital care produces a high carbon footprint (Rodriguez-Jimenez et al., 2023). ESD is also shown to be beneficial regarding the patients' functioning, in terms of independency in daily activities early after stroke (Bjorkdahl et al., 2023). Thereby, ESD contributes to three principles of sustainable healthcare; lean pathways in that it involves effective rehabilitation and is resource saving, and empowerment and prevention in that ESD implements rehabilitation strategies applied by patients directly in their home environment, which also involves preventive strategies to reduce future healthcare consumption (Figure 2). Another example is the use of exoskeletons in upper limb rehabilitation, highlighting the need for sustainability evaluation due to the particularly high monetary costs of the equipment (Pinelli et al., 2023). The authors propose the usefulness of a sustainability evaluation including economic, social and environmental values as a framework to ensure sustainability in clinical decision-making in stroke rehabilitation (Pinelli et al., 2023). When it comes to the importance of dose specific rehabilitation, it has been found difficult to achieve the intervention dose needed to improve functioning, for example in upper limb rehabilitation after stroke (Hayward et al., 2021). This implies that an insufficient dose of rehabilitation could be a waste of valuable resources, hence not contributing to sustainable healthcare. Rather, an adequate rehabilitation dose is essential for achieving sustainable healthcare.

The second key aspect of how multidisciplinary stroke team rehabilitation contributes to sustainable healthcare is through the support of self-efficacy and patient empowerment. Stroke team rehabilitation interventions focusing on self-management, such as training in activities of daily life (ADL) or other task-oriented interventions, have been found not only to increase self-efficacy but also to be beneficial for quality of life and health status after stroke (Jones and Riazi, 2011). Self-efficacy shapes health behaviors by influencing the goals that individuals set, the effort they put into achieving those goals, and their perseverance in the face of challenges or setbacks (Dixon et al., 2007). By nature, rehabilitation involves empowerment of patients for strengthening of capacities and promoting self-management. This directly supports the principle of *empowerment* but also aligns with all other principles of sustainable healthcare. It aids in *prevention* by equipping patients with tools to maintain their health status, promotes *lean pathways* by reducing the need for additional healthcare interventions, and supports *low carbon alternatives* and *operational resource use* due to its resource efficiency (Figure 2).

The third significant aspect of how multidisciplinary stroke team rehabilitation contributes to sustainable healthcare concerns the use of telerehabilitation as a low resource delivery mode. Digital health involves using digital technologies to enhance health, including telerehabilitation, which delivers rehabilitation through information and communication technologies (World Health Organization, 2021). Telerehabilitation interventions have been shown to be cost-effective (Jiang et al., 2019) and could also be considered a *low carbon alternative* with a *low operational resource use* (Figure 2) compared to clinical visits, as a means for reducing the environmental burden of healthcare, where substituting clinical visits with digital health could lead to fewer transports and hence less vehicle emissions (Masino et al., 2010; Purohit et al., 2021). Telemedicine has been found to significantly reduce carbon emissions, with savings ranging from 0.70 to 372 kg CO_2_e per consultation, primarily due to reduced travel, while the systems themselves produce minimal emissions (Purohit et al., 2021). A review found moderate evidence for the effectiveness of digital health on motor function, activities of daily living, independence, satisfaction and quality of life in patients with stroke (Appleby et al., 2019). However, telerehabilitation requires resources and infrastructure which need to be considered (Purohit et al., 2021), and the accessibility to technology in patients should be considered when using telerehabilitation in stroke team rehabilitation.

The fourth crucial aspect of how multidisciplinary stroke team rehabilitation contributes to sustainable healthcare involves secondary prevention and health promotion, which minimize the need for additional care. After a first stroke or Transient Ischemic Attack (TIA), effective secondary prevention could reduce the burden of stroke by almost 25% (Hankey, 2014). Secondary prevention is therefore an important part of stroke management and important for sustainable stroke care, including the work conducted by all team members. Ten modifiable risk factors cause 90% of stroke incidences, there among physical inactivity, hypertension, unhealthy diet, smoking, and excessive alcohol intake (O'Donnell et al., 2016). Therefore, addressing modifiable risk factors for stroke is key in secondary prevention, commonly including counseling, patient education, risk factor management, and supervised exercise provided by health professionals in the stroke team (Liljehult et al., 2020). Improved health behavior is widely recommended to be included in secondary prevention (Kernan et al., 2014) and modifiable risk factors need to be managed not only with pharmacological treatment, but also with health behavior change (Boehme et al., 2017). Interventions focusing on secondary prevention in stroke through health behavior change have found effects on behavioral risk factors, blood pressure, as well as cardiovascular events (Liljehult et al., 2020). Clearly, secondary prevention contributes to the first principle of sustainable healthcare, which is *prevention*, but it also supports all other principles. It *empowers* patients by providing them with knowledge and tools for self-management to maintain their health and prevent further disease. Additionally, secondary prevention promotes *lean pathways* by reducing the need for additional healthcare resources through disease prevention. Moreover, preventive strategies for stroke are *low in carbon emissions* and *operational resource use* compared to the resource-intensive care required during stroke treatment, thereby helping to reduce the carbon intensity of care.

## Path forward

Multidisciplinary stroke team rehabilitation is not only considered best practice in stroke care but is also essential for achieving sustainable stroke care. It optimizes functioning which has numerous co-benefits for healthcare resource use and contributes to all principles for sustainable healthcare; prevention, patient empowerment, lean pathways, low carbon alternatives and efficient operational resource use. First, timely assessment and dose specific interventions are essential for successful stroke rehabilitation outcomes and are a central focus of the multidisciplinary stroke team rehabilitation aimed at optimizing patient functioning. Providing team rehabilitation at the right time and in the right dose to the patients who benefit most will optimize functioning and, in addition, yield important co-benefits for sustainable healthcare. Achieving beneficial rehabilitation outcomes hinges on providing an adequate dose, which can be challenging in a clinical setting. Nevertheless, investing in a sufficient intervention dose is crucial to avoid wasting valuable patient and staff resources. While this may require greater short-term resource allocation, it will ultimately lead to optimized functioning, improved quality of care, and reduced overall healthcare resource use, thereby contributing to sustainable healthcare.

Second, self-efficacy and patient empowerment are recognized as core values in rehabilitation relating to patient autonomy. By enhancing self-management and empowering patients, healthcare utilization can be reduced without compromising health outcomes (Panagioti et al., 2014), thereby contributing to more sustainable healthcare over time. Third, telerehabilitation, as an alternative to physical healthcare visits, holds great potential for people with stroke, particularly in increasing accessibility to healthcare services where transportation is challenging. The benefits of telerehabilitation extend beyond reduced travel, offering increased access to neurorehabilitation by complementing hospital-based procedures with telerehabilitation (Brennan et al., 2021). Last, preventive healthcare activities make a dual contribution to sustainable stroke due to their very low carbon footprint compared to more resource-intensive healthcare activities such as hospital or ambulatory care (Pichler et al., 2019). But foremost, there are vast social, environmental and economic benefits from stroke prevention relating to the potential decrease in years lived with disability caused by stroke (Cieza et al., 2021) along with the tremendous resources used in hospital care due to stroke (Lekander et al., 2017; Strilciuc et al., 2021) and the environmental impact from hospital emissions (Rodriguez-Jimenez et al., 2023). These aspects combined provide a strong incentive for optimizing secondary prevention in stroke. Additionally, health behavior changes often have environmental co-benefits; for example, dietary changes that are beneficial to health also have a lower carbon footprint (Crippa et al., 2021). Similarly, integrating more physical activity into daily life can lead to reduced greenhouse gas emissions due to decreased transportation needs (Quam et al., 2017). The significant contribution of secondary prevention to sustainable stroke care should be acknowledged, as it reduces stroke recurrence and thereby minimizes the need for care.

As demonstrated, multidisciplinary stroke team rehabilitation aligns with all principles of sustainable healthcare. Utilizing a sustainability framework to analyze healthcare activities allows us to identify both sustainable and less sustainable practices (Mortimer et al., 2018). Generally, striving for sustainable healthcare involves reducing healthcare activities (Mortimer et al., 2018). However, in the case of stroke team rehabilitation, the opposite is true. Increased investment in rehabilitation resources leads to lower overall healthcare resource use. This is because rehabilitation interventions typically have a low carbon footprint and result in optimized functioning and health, thereby reducing overall healthcare consumption over time.

## Future directions

Evaluations of stroke rehabilitation interventions that consider environmental, economic, and social aspects of sustainability are scarce. We strongly recommend that future evaluations of stroke rehabilitation interventions incorporate sustainable development perspective. This approach will uncover the contribution of multidisciplinary stroke team rehabilitation to sustainable healthcare.

## Data Availability

The original contributions presented in the study are included in the article/supplementary material, further inquiries can be directed to the corresponding author.
